# CSF biomarkers of B-cell activation in multiple sclerosis: a clinical perspective

**DOI:** 10.1007/s00415-025-12907-6

**Published:** 2025-02-17

**Authors:** Elena Di Sabatino, Diana Ferraro, Lorenzo Gaetani, Edoardo Emiliano, Lucilla Parnetti, Massimiliano Di Filippo

**Affiliations:** 1 Clinica Neurologica, Dipartimento di Medicina e Chirurgia, Università di Perugia, Perugia, Umbria Italy; 2 Dipartimento di Neuroscienze, Ospedale Civile di Baggiovara, Azienda Ospedaliera-Università di Modena, Modena, Italy

**Keywords:** Multiple sclerosis, Fluid biomarkers, B cell, Oligoclonal bands, κ-index, BAFF/APRIL

## Abstract

The role of B cells in the pathophysiology of multiple sclerosis (MS) extends beyond antibody synthesis, also involving the modulation of T lymphocytes and myeloid cells. B-cell activation within the Central Nervous System is associated with the release of various antibodies, cytokines, and chemokines, measurable in biofluids, thereby serving as biomarkers of the immune processes responsible for MS. To this purpose, a biomarker-based characterization of the disease through the combination of well-established markers, e.g., immunoglobulin (Ig) G index, IgG oligoclonal bands, Ig free light chains, with new promising markers, namely chemokine (C–X–C motif) ligand 13, and B-cell activating factor/A proliferation-inducing ligand, might represent a significant improvement in the management of people with MS.

## Introduction

Multiple sclerosis (MS) is the most prevalent chronic immune-mediated, inflammatory, and neurodegenerative Central Nervous System (CNS) disease in young adults [1, 2].

The pathological hallmark of MS is the presence of focal areas of inflammatory demyelination around post-capillary venules throughout the CNS, characterized by the breakdown of the blood-brain barrier (BBB), with subsequent infiltration of macrophages, T cells, and B cells. Recurrent waves of peripheral autoreactive lymphocytes infiltrate the CNS especially in the early phases of MS causing structural and functional disconnection of CNS areas. Axonal loss and neurodegenerative processes are evident since the disease onset and, over time, dominate MS pathology, contributing to the accumulation of irreversible clinical disability. This progression is sustained by the failure of the cerebral functional reserve combined with the persistence of a “smouldering” inflammatory process within the CNS [3]. Such compartmentalized inflammation involves an abnormal intrathecal immune activation of both resident innate immune cells, such as microglia and astrocytes, and  adaptive immune cells, particularly B cells clustered in ectopic lymphoid structures in cerebral meninges [2, 4, 5].

From a clinical perspective, MS is a heterogeneous disease. The MS spectrum ranges from a pre-clinical phase, often discovered in asymptomatic subjects through incidental magnetic resonance imaging (MRI) (a condition referred to as radiologically isolated syndrome [RIS]) [6], to a clinical phenotype where recurrent acute/subacute episodes of neurological dysfunction (i.e., relapses) are accompanied by the slow development of permanent and progressive neurological disability independent of relapses (progression independent of relapse activity [PIRA]) [7, 8].

Diagnosis relies on demonstrating the presence of focal inflammatory lesions in at least two distinct typical anatomical locations within the CNS (i.e., dissemination in space [DIS]) at different time points (i.e., dissemination in time [DIT]) and on excluding alternative diagnoses [9]. DIS and DIT as well as the exclusion of alternative, better explanation, are established through the combination of the clinical picture and paraclinical exams, such as CNS MRI and cerebrospinal fluid (CSF) analysis. Among them, CSF analysis stands as an informative tool, helpful in ruling out infectious conditions and in demonstrating the inflammatory etiology through the evidence of intrathecal immunoglobulin G (IgG) synthesis. The latter is also utilized as an alternative criterion for the demonstration of DIT in the currently used MS diagnostic criteria [9]. For these reasons, CSF analysis allows for an accurate and early diagnosis, leading to the prompt initiation of disease-modifying therapies (DMTs) with the aim of reducing disease activity and delaying the progression of MS [10]. It is worth noting that a revision of MS diagnostic criteria (presented at the latest congress of the European Committee for Treatment and Research in MS [ECTRIMS], Copenhagen, 2024) is expected.

Within MS research and clinical practice, several critical questions remain unanswered on the path toward the optimization of personalized treatments capable of halting disease progression: staging the disease, predicting the disease course, intercepting disease progression, and accurately monitoring treatment response [2, 4]. In this scenario, fluid biomarkers reflecting the disease pathophysiology hold promise for these purposes [11].

**Fig. 1:**
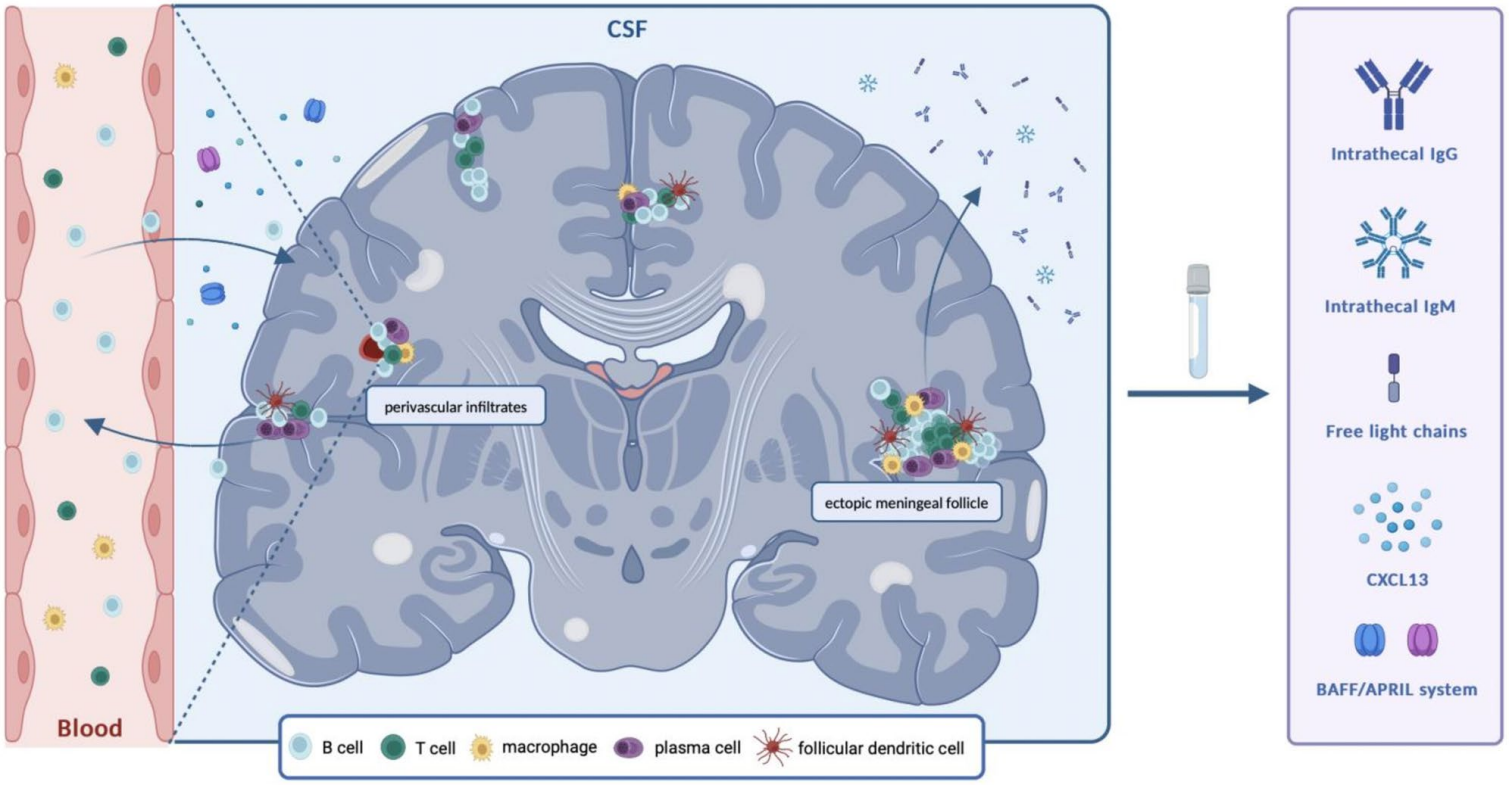
**CSF biomarkers of intrathecal B-cell activation in MS.** Autoreactive B cells enter the CNS via post-capillary venules and contribute to the development of demyelinating plaques. In addition, B cells form meningeal aggregates supporting the CNS-compartmentalized inflammation. Clonally expanded, terminally differentiated B cells in the CNS compartment produce immunoglobulins (IgG and IgM) as well as free light chains. The migration and activation of these B cells within the CNS require the presence of chemokines and cytokines, such as CXCL13 and the BAFF/APRIL system. An ongoing exchange of B cells between the CNS and peripheral blood is crucial for maintaining intrathecal B-cell stimulation. Immunoglobulins and free light chains production, as well as increased levels of chemokines/cytokines in CSF, might be the quantifiable epiphenomenon of B-cell activation within the CNS and, thus, could be measured as biomarkers to reveal immune processes responsible for MS and to improve the management of pwMS. Abbreviations: CSF: cerebrospinal fluid; MS: multiple sclerosis; CNS: Central Nervous System; CXCL13: C–X–C motif chemokine ligand 13; BAFF: B-cell activating factor; APRIL: A proliferation-inducing ligand; pwMS: people with MS

With consistent data highlighting the key role of B cells in MS pathophysiology [12], our understanding of the disease has evolved. This review aims to explore the current knowledge on CSF biomarkers of B-cell activation in the CNS, which is associated with the release of various antibodies, cytokines, and chemokines, measurable in biofluids, mirroring the immune processes responsible for MS, and holding diagnostic and prognostic value in MS management. Both markers of B-cell activation already used in clinical practice such as IgG index and IgG oligoclonal bands (OCGBs) and promising markers, such as immunoglobulin free light chains (FLC), immunoglobulin M (IgM) index, and IgM oligoclonal bands (OCMBs), together with C–X–C motif ligand 13 (CXCL13), and B-cell activating factor (BAFF)/A proliferation-inducing ligand (APRIL) are discussed (Fig. 1).

## B cells and MS pathophysiology: what do we know?

The historical view of MS as a T-cell-mediated disease, involving an abnormal balance between regulatory T cells (T_reg_) and CNS-reactive effector T cells (T_eff_), has undergone significant evolution in recent years, with mounting evidence implicating the crucial involvement of B cells [12].

B lymphocytes, traditionally associated with humoral immune responses through antibody secretion, have been shown to own additional, antibody-independent functions such as antigen presentation and cytokine production, with both stimulating and modulating implications on other immune cell responses. [12, 13]. B cells are able to enhance and sustain T CD4^+^ responses by acting as antigen-presenting cells (APCs) [13–15]. On the other hand, B-cell populations capable of down-regulating immune responses, referred to as regulatory B cells (B_reg_), have been identified, acting in a similar way to T_reg_ cells and enhancing, in turn, T_reg_ responses [16–18].

B-cell responses play a key role in inflammatory and degenerative MS processes through both antibody-related and -independent mechanisms in the periphery and within the CNS [12]. The involvement of B cells in MS pathogenesis is supported by evidence from neuropathology and fluid biomarkers in people with MS (pwMS) and in animal models such as experimental autoimmune encephalomyelitis (EAE) [12].

Neuropathological studies have shown antibody deposition and complement activation in many demyelinating lesions [19] and B-cell infiltrates within the CNS. The latter are predominantly found in the perivascular spaces and meninges, forming tertiary lymphoid structures, an important site of immune cell activation and expansion. These meningeal B-cell-rich aggregates release immunoglobulins, cytokines, and soluble toxic factors, thus supporting the CNS-compartmentalized inflammation which plays a crucial role in propagating the constant CNS injury underlying progressive phases of the disease [12, 20]. As an example, meningeal B-cell follicle-like structures have been identified mainly in people with progressive MS, in whom their presence correlates with the severity of neuron and oligodendrocyte loss in the cortex and with clinical disability [21, 22].

Another significant clue of a role of B cells in the pathogenesis of the disease is the observed increased production of immunoglobulins in the CSF, which can be detected in the form of OCGBs in most pwMS [23]. The target antigens of these antibodies, which remain largely unknown, include viral antigens, particularly those associated with Epstein–Barr virus (EBV), myelin antigens, and ubiquitous intracellular antigens [24–29]. These findings indicate that the B-cell response in MS is heterogeneous and partly directed against autoantigens released during tissue destruction [27]. Besides immunoglobulins, within the CNS, there is a local production of chemotactic factors for B cells (such as CXCL10, CXCL12, and CXCL13), which support homing, survival, and functional activation of B cells [30, 31]. Interestingly, it has been recently demonstrated, through somatic hypermutation studies, that clonally related B lymphocytes act as immunologically active links between the periphery and CNS, and that OCGBs-producing B cells are present also in the blood [32]. In fact, B cells involved in OCGBs expression can move from the CNS to the peripheral blood, where further affinity maturation may occur. This suggests that OCGBs are not just the endpoint of a targeted immune response in MS, but rather a component of active B-cell immunity operating on both sides of the BBB [33].

The presence of intrathecal synthesis of immunoglobulins was one of the bases for testing anti-B-cell therapies, such as anti-CD20 monoclonal antibodies, which proved to be among the most effective approved therapies in MS. [34]. Interestingly, however, evidence supports that the role of B cells in the pathogenesis of MS is also linked to their antibody-independent features [35–37]. The antibody-independent functions of B cells in MS have been studied in EAE models, where results confirmed the dual role of these cells in sustaining and down-regulating immune responses. The function of B cells as APCs involved in the induction of EAE was demonstrated in myelin oligodendrocyte glycoprotein (MOG)-induced EAE models, in which the selective knock-out of class II major histocompatibility complex (MHC) on B cells counteracted disease induction [38]. On the other hand, EAE mice models depleted of B cells before EAE induction exhibited a more aggressive disease phenotype, indicating a role for these cells in negatively modulating immune responses involved in the pathogenesis of neuroinflammation [39]. Moreover, the intravenous administration of B_reg_ cells in EAE mice produced complete EAE remission and promoted remyelination and oligodendrogenesis, through the activation of T_reg_ cells in secondary lymphoid organs and their subsequent migration into the CNS [40, 41].

In summary, all these data have led toward a complex paradigm of MS immune pathophysiology where the constant and bidirectional interactions between B cells, T cells, and myeloid cells in the periphery and microglia in the CNS play an essential role in both the inflammatory and neurodegenerative aspects of the disease.

## Fluid biomarkers of immunoglobulin production

The most prominent immunological abnormality in MS is the presence of elevated immunoglobulin concentration in the CSF, a long-standing disease hallmark. From a pathophysiological point of view, debates persist on the role of intrathecal immunoglobulins, questioning whether they actively contribute to CNS inflammation or merely serve as markers of ongoing inflammation [28]. Among immunoglobulins, IgG is the most commonly assessed isotype. However, other immunoglobulin isotypes (IgM, IgA) also show distinctive increases in MS CSF compared to blood [42, 43].  Elevated levels of intrathecal immunoglobulins can be evaluated with quantitative (CSF/serum quotient diagrams with or without hyperbolic reference range, such as the Reibergram and Ig index) and qualitative tests (determination of oligoclonal bands [OCBs]) [44–46]. The gold standard method is identifying CSF-restricted OCBs through isoelectric focusing (IEF) [23, 45]. An excess of immunoglobulin kappa and lambda FLC can also be produced during chronic intrathecal inflammation; they can be measured rapidly and quantitatively, and kappa FLC (KFLC) in particular has gained significant interest in the last years [47, 48].

### IgG intrathecal synthesis

IgG synthesis in the CSF, as measured by means of OCGBs, is among the strongest indicators that an antigen-driven humoral immune process contributes to MS pathology. The qualitative demonstration of two or more CSF-specific OCGBs indeed reliably indicates intrathecal antibody synthesis. They currently serve as the most established and widely used molecular biomarker in clinical practice for the diagnostic work-up of MS [49].

OCGBs reflect the synthesis of soluble clonal IgG with restricted heterogeneity [33], which are produced by clonally expanded, terminally differentiated B cells in the CNS [50, 51], suggesting a highly targeted immune response against yet unknown antigens [44, 52]. It is likely that within perivascular infiltrates and meningeal lymphoid-like follicles in the CNS, B cells undergo antigen-directed affinity maturation and differentiate into antibody-producing plasma cells [21, 53].

Methodologically, agarose gel electrophoresis with IEF followed by immunoblotting or immunofixation for IgG is the recommended technique for detecting OCGBs, involving the analysis of paired, undiluted CSF and serum samples, to confirm that the OCGBs are unique to CSF or prevalent in the CSF. However, IEF is time-consuming, and technical challenges as well as operator-dependent pattern interpretations can impact reproducibility and result accuracy [46].

OCGBs are present in up to 95% of pwMS, with a prevalence ranging between 68% and 83% already at the first clinical manifestation of the disease [53, 54]. The presence of CSF OCGBs represents a central immunodiagnostic feature for MS, supporting the diagnosis in combination with other clinical and imaging criteria. Particularly, in the context of relapsing MS, the 2017 revision to the McDonald criteria allows the presence of CSF OCGBs to substitute for the requirement of fulfilling dissemination in time (DIT) through clinical or MRI data, to reach an early diagnosis [9]. Indeed, in a study on 137 pwMS, it was demonstrated that, by defining DIT also in the presence of CSF OCGBs, time to diagnosis was shortened and anticipated by around one year [55]. This is grounded on the observation that in individuals at the first clinical manifestation of the disease, CSF OCGBs are an independent predictor of the risk of a second attack [54, 56]. The demonstration of CSF OCGBs can increase the diagnostic confidence also in the context of a primary progressive course [9]. The role of OCGB determination in the forthcoming revision of MS diagnostic criteria, as presented at the latest ECTRIMS congress, will soon be revealed.

However, OCGBs are not exclusive to MS and can also be found in various other inflammatory neurological conditions [57]. A comprehensive meta-analysis on the intrathecal IgG synthesis in MS, including 13,467 subjects, underlines how, despite the high-test sensitivity, the diagnostic specificity of OCGBs drops from 94 to 61% if alternative inflammatory diseases are considered in the differential diagnosis [57]. Furthermore, approximately 5% of MS cases do not show CSF OCGBs [46]. Interestingly, as hypothesized in a study including 118 individuals (58 OCGB + pwMS, 24 OCGB- pwMS, and 36 subjects with other neurological diseases), the absence of CSF OCGBs does not definitively exclude an MS diagnosis, but it might point to a specific subtype of the disease characterized by a distinct immunological profile in the CSF [58], and consequently a different underlying pathophysiological substrate.

Beyond their supportive diagnostic role, OCGBs serve as a prognostic indicator for future disease activity and course [59, 60]. OCGBs have been linked to a faster conversion from RIS to clinically definite MS [61], and to a higher probability of reaching certain specific disability milestones. For instance, in a study on 1000 pwMS at the early stage, followed for 81 months, OCGB-positive subjects had a twofold higher risk for achieving the Expanded Disability Status Scale (EDSS) score ≥ 3.0, nearly as high as the risk conferred by having 10 or more MRI lesions [56]. Tur and colleagues, in a longitudinal study aiming at investigating clinical, laboratory, and neuroimaging predictors of PIRA at the time of the first demyelinating attack in a cohort of 1128 subjects, found that individuals with subsequent evidence of PIRA were more prone to having CSF OCGBs than those without PIRA [62]. Additionally, OCGBs correlate with worse cognitive performance in pwMS. It has been demonstrated that OCGB-positive pwMS more often exhibit cognitive impairment compared to OCGB-negative pwMS and, when evaluating a single cognitive domain, OCGBs were found to specifically correlate with memory impairment [63]. This aligns with findings indicating that the presence of CSF OCGBs is linked to a higher load of cortical lesions on brain MRI [64], potentially responsible for impaired cognitive functions such as memory [65].

Taken together, the prognostic effect of OCGBs and their correlation with different disease severity measures provide evidence that they may reflect more active CNS-directed autoimmunity and more intense compartmentalization of immune activity into tertiary lymphoid structures releasing inflammatory mediators that favor neurodegeneration [22]. As a proof of this, the occurrence of OCGBs correlated with higher levels of neurofilament light chain (NfL), a robust biomarker of disease intensity and acute axonal damage, and increased inflammatory mediators linked to B-cell activity and lymphoid neogenesis [64]. However, it is noteworthy that, although many studies have found a possible predictive power of CSF OCGBs in MS, several have shown that the lack of OCGB positivity does not necessarily ensure a more favorable disease course [66].

Finally, OCGBs provide only a qualitative, and not quantitative, information about intrathecal B-cell activation [46]. A quantitative measure of intrathecal IgG production, such as IgG index (CSF/serum IgG ratio divided by CSF/serum albumin ratio), is less diagnostically sensitive than OCGBs and more prone to false-positive results in the presence of BBB damage [67]. A prognostic effect of the IgG index has been demonstrated with elevated levels correlating with worse clinical course, increased risk of conversion to progressive MS [68], and the severity of cerebral gray matter atrophy [69]. However, the low diagnostic accuracy of the IgG index limits its value in clinical routine.

### Intrathecal IgM synthesis

The occurrence of intrathecal IgM synthesis has been documented in 20–55% of pwMS, a phenomenon often associated with an unfavorable prognosis [42, 70]. Unlike peripheral blood IgM, intrathecal IgM in pwMS exhibits distinctive traits, including the lack of class switching to IgG and a high degree of somatic hypermutation [71]. Moreover, in MS, intrathecal IgM synthesis persists, suggesting an enduring immune response, rather than an acute phase phenomenon, as it occurs in infections [72]. Although debated, IgM might play a pathogenic role, since pathological studies have identified antibody-mediated demyelination in certain MS lesions (the so-called “pattern II lesions”) [73]. In these lesions, both IgG and IgM co-localize on oligodendrocytes and axons with complement and foamy macrophages. Notably, the multimeric structure of IgM antibodies makes them potent complement activators, leading to more pronounced demyelination and axonal damage [74]. Moreover, in animal models, IgM directed against glycolipids induced CNS demyelination and prevented remyelination [75].

In assessing intrathecal IgM synthesis, quantitative and qualitative methods can be employed. As a qualitative method, detecting OCMBs poses technical challenges due to IgM's hydrophobic nature, lower CSF concentrations compared to IgG, and in vivo aggregation in pentameric structures. A new technique involving IgM reduction at alkaline pH and a highly sensitive IEF method has revitalized OCMBs detection [76]. Furthermore, recent research indicates good interlaboratory reproducibility [77, 78]. As quantitative methods, both IgM index (CSF/serum IgM ratio divided by CSF/serum albumin ratio) and IgM intrathecal fraction (IgM_IF_) can be used [42, 79, 80].

From a clinical standpoint, the evidence of intrathecal IgM synthesis carries significant prognostic implication. A systematic review and meta-analysis pooling data from 30 studies, encompassing 5000 pwMS, revealed an overall prevalence of intrathecal IgM synthesis (detected with either qualitative or quantitative methods) of 29%. Additionally, within this work, a meta-analysis focusing on a subset of 1221 early relapsing MS cases found that subjects with intrathecal IgM synthesis faced a markedly elevated risk of a subsequent clinical relapse, surpassing the risk associated with OCGBs [42]. Furthermore, several studies have linked intrathecal IgM synthesis not only to heightened susceptibility to relapses [81, 82], but also to an increased disability [83], a shorter time to progression onset [84], and a more inflammatory phenotype in pwMS presenting a primary progressive course [85]. Recent research has further underscored the prognostic significance of intrathecal IgM synthesis using quantitative methods. For instance, Rosenstein et al., in a study including 457 pwMS, demonstrated that both IgM index and IgM_IF_ serve as useful biomarkers in early relapsing MS, by predicting future disease activity over a 2-year follow-up period (outperforming OCMBs), and future disability [86]. Another study found that the presence of IgM_IF_ in 122 individuals experiencing their first demyelinating event is independently associated with spinal lesions, which are known to be strong predictors of physical disability [87].

The specificity of OCMBs in MS has also been investigated, revealing that a considerable percentage of OCMBs recognize specific myelin lipidic antigens, termed lipid-specific OCMBs (LS-OCMBs) [88]. The presence of CSF LS-OCMBs at MS onset correlates with a more aggressive disease course compared to total OCMBs [89], increased disability in long-term follow-up [90], early rise in lesion load and brain atrophy [91], elevated CSF NfL [92], and increased retinal axonal loss evaluated via optical coherence tomography [93].

### Immunoglobulins free light chains and kappa-index

OCBs are limited by the fact that they do not provide a quantitative measure of immune activation; instead, they merely represent a qualitative outcome [46]. To overcome this limitation, other quantifiable markers of immunoglobulin intrathecal synthesis are available (such as IgG and IgM indices, or IgM_IF_)_,_ though limited by lower accuracies as in the case of IgG index compared to OCGBs. Further, all the above-mentioned quantitative measures are immunoglobulin isotype-specific, reflecting only a part of humoral immunity activation in the CNS.

Immunoglobulins are formed by two heavy chains, which determine their class (IgG, IgM, IgE, IgD, and IgA) and by two light chains (either kappa or lambda). FLC are observed in the circulation due to the 10 to 40% excess synthesis of light over heavy chains in plasma cells. FLC in sera and CSF increase in inflammatory diseases, presumably as a result of B-cell activation [47].

The intrathecal synthesis of FLC, and in particular of KFLC, is gaining increasing interest as a marker of intrathecal immunoglobulin synthesis [48]. KFLC can be easily measured by nephelometry or turbidimetry, which are automated, less costly and less time-consuming methods compared to CSF OCBs detection using IEF, and the read-out is, furthermore, quantitative and not operator-dependent [94–97].

The intrathecal KFLC synthesis can be calculated using different metrics, including the linear kappa (κ)-index (CSF/serum KFLC ratio divided by CSF/serum albumin ratio) and the KFLC intrathecal fraction, which takes the non-linear relationship of the blood-to-CSF transfer between albumin and KFLC into account [98]. The most utilized metric is the κ-index, and there is a growing body of evidence indicating that an elevated κ-index is a more sensitive marker of intrathecal immunoglobulin synthesis compared to the detection of CSF-restricted OCGBs, showing a similar specificity.

The κ-index has been extensively studied in the diagnostic work-up of MS. A recent systematic review and meta-analysis, which included a total of 32 studies involving 3,322 pwMS and 5,849 controls, identified an optimal cut-off of 6.1 in predicting an MS diagnosis and demonstrated a similar diagnostic accuracy compared to OCGBs detection. Average sensitivity and specificity were 88% and 89% for the κ-index and 85% and 92% for OCGBs [99]. Of note, the accuracy of κ-index is similar to that of OCGBs even in the context of primary progressive MS, where the evidence of intrathecal IgG synthesis is a relevant criterion for the diagnosis [100]. Based on the consistently high diagnostic accuracy of the κ-index in identifying pwMS across studies, it was already suggested to include the κ-index in the revision to the 2017 McDonald diagnostic for MS [101–103]. Due to its greater sensitivity, the κ-index can be particularly useful, in the appropriate clinical context, in patients with suspected MS and absent OCGBs, or with a single CSF IgG band. An elevated κ-index (≥ 5.8—6.4) was found in 25–54% of OCGB-negative pwMS [104–106] and in 38–43% of individuals with a single CSF IgG band [106, 107]. KFLC synthesis determinations, as an additional tool to support the diagnosis of MS, will be potentially included, as an alternative to OCGBs detection, in the next revision of MS diagnostic criteria (announced at the latest ECTRIMS congress, Copenhagen, 2024).

Another added value of the κ-index and its quantitative nature is its possible prognostic role in predicting disease activity, as demonstrated in the recent studies [108–111]. In a study by Berek et al., involving a cohort of 88 pwMS, individuals with a κ-index > 100 at baseline had a median time to future relapse of 11 months compared to 36 months in pwMS with a κ-index ≤ 100 [110]. Similarly, the chance for freedom from relapse within 12 months from disease onset was 2% in pwMS with a κ-index > 100 and a high serum NfL Z score (> 3), and 90% in pwMS with a κ-index ≤ 100 and a low serum NfL Z score (≤ 3) [108]. Another study on 182 subjects provided evidence that the κ-index predicts new T2 lesions in individuals at the earliest disease phases (both RIS and the first clinical manifestation) [111]. Finally, data from the Swedish MS registry on 131 pwMS at early stage showed that the κ-index was significantly higher in individuals with PIRA over a period of 4 years, and that people with a κ-index > 100 had an almost fourfold increase in the risk for developing PIRA [109].

Ultimately, κ-index is associated with worse cognitive performance in pwMS [112]. In particular, recent research, conducted on 39 newly diagnosed pwMS, showed that κ-index negatively correlates with verbal memory, independently of MRI measures of disease severity (such as brain lesion load, brain volume, and brain regional volumes). This finding suggests a potential direct negative effect of intrathecal activated B cells on cortical functions, disrupting brain network functioning beyond observable structural damage on MRI scans [113].

## Cytokines and chemokines reflecting B-cell activity

In addition to biomarkers indicating intrathecal immunoglobulin synthesis, the assessment of B-cell activity extends to the measurement of soluble mediators in the CSF. These mediators, released by various immune cells, play a crucial role in facilitating the migration (chemokines) and activation (cytokines) of B cells within the CNS. These markers might allow for a more comprehensive assessment of B-cell activity than markers of intrathecal immunoglobulin synthesis.

### CXCL13

CXCL13, a small (10.3 kDa) chemokine primarily produced by follicular dendritic cells and stromal cells within the lymphoid follicles, serves as a major chemoattractant, by binding to the CXCR5 receptor, for B cells and follicular T helper cells [114]. It is responsible for the organization of B cells in the lymphoid follicle in secondary lymphoid organs and, in turn, in the MS landscape, it is involved in B-cell maturation and migration, being thus essential for the formation and maintenance of ectopic meningeal B-cell follicles [53].

CXCL13 can be measured in CSF only or in CSF and serum to obtain a CXCL13 index (CSF/serum CXCL13 ratio divided by CSF/serum albumin ratio). However, the superiority of CXCL13 index over CSF CXCL13 is debated, since evidence argues against the crossing of BBB of CXCL13 from the periphery to the CNS [115]. In measuring CXCL13 levels, ELISA has been the predominant methodology used, despite its sensitivity challenges. Other more sensitive methodologies have been used, including single molecule array (Simoa) and Luminex assay, but these are not readily available for most clinical laboratories [116].

Elevated levels of CXCL13 have been consistently observed in the CSF of pwMS [117–119]. As to specificity, however, CXCL13 intrathecal production is not exclusive to MS, as it is seen also in other inflammatory or infectious diseases, particularly in neuroborreliosis [120]. Thus, the utility of CXCL13 as a diagnostic biomarker for demyelinating disease is limited.

On the other hand, in MS high CSF CXCL13 concentrations have been found to be associated with the risk of a second relapse in individuals at the first clinical manifestation [121, 122]. Indeed, CXCL13 index is especially helpful in predicting future disease activity as defined by attacks or new or enhancing MRI lesions, even with better accuracy than OCGBs (CXCL13 index: sensitivity/specificity, 91% [95% CI = 69–98%] / 64% [95% CI = 43–81%]; OCGBs: sensitivity/specificity, 81% [95% CI = 60–93%] / 30% [95% CI = 16–49%]) [123], potentially providing additional information to biomarkers of intrathecal IgG synthesis. In relapsing MS, high CSF CXCL13 concentrations have been found to be correlated with increased relapse rates and disease severity measured by EDSS [121]. Moreover, elevated CSF CXCL13 levels specifically predicted rapid disease progression, correlating with higher cortical lesion load in pwMS [30].

### BAFF/APRIL system

BAFF and APRIL, a structural homologue of BAFF, are pleiotropic inflammatory cytokines, primarily produced by monocytes and neutrophils, particularly involved in B-cell activation, differentiation, and survival. BAFF exists in membrane-bound and secreted, biologically active, soluble forms after cleavage at a furin protease site, while APRIL is mainly produced in a soluble form [124]. While these cytokines play essential roles in the physiological regulation of B-cell immunity, their inappropriate production has been implicated as a key factor disrupting immune tolerance, contributing to the pathogenesis of several immune-mediated disorders, including MS [125]. A genetic variant in TNFSF13B, encoding BAFF, causing its abnormal expression and leading to an excess of B-cell activation, has been associated with MS as well as with systemic lupus erythematosus [126]. Immunohistochemistry reveals constitutive BAFF presence in the CNS and up-regulation in demyelinating lesions [127]. These cytokines are also being explored as potential treatment targets due to their role in the underlying disease pathogenesis. Interestingly, mechanistically different MS treatments converge in increasing serum BAFF while decreasing memory B cells, the latter known as disease-promoting [128–130]. This is in line with the fact that, despite drugs targeting the BAFF/APRIL system such as atacicept have been adopted for the treatment of other autoimmune disorders, to date they provided unsatisfactory results in MS [125]. In particular, CNS inflammatory disease activity resulted elevated rather than diminished with these treatments, potentially disrupting the balance of B-cell subsets and regulatory pathways. For instance, atacicept preferentially limits the survival of naïve B cells and plasmablasts or plasma cells, but has a lesser effect on memory B cells [125, 131, 132].

Various studies evaluated CSF BAFF/APRIL levels in MS, measured through ELISA or Luminex assays, without conclusive results, even though it is worth noting that these methodologies may lack the sensitivity required to detect subtle changes in biomarker levels. Some studies demonstrated elevated BAFF and APRIL in CSF, but not in serum, of pwMS compared to healthy individuals [133]. This finding emerged also in other infectious diseases such as neuroborreliosis [134], or immune-mediated diseases of the CNS, such as neuromyelitis optica spectrum disorder [135] and MOG antibody-associated disease [136], indicating BAFF as a potential determinant of B-cell activity in inflammatory CNS diseases.

Several studies on intrathecal MS inflammation found increased levels of both BAFF and APRIL in the CSF of a subgroup of naïve pwMS who, at time of diagnosis, were characterized by a high cortical lesion load and high CSF levels of other inflammatory mediators linked to B-cell recruitment and activity as well as to lymphoid neogenesis. A similar intrathecal inflammatory milieu was also found in post-mortem progressive MS cases characterized by the presence of elevated meningeal inflammation, cortical demyelination and more rapid and severe disease progression [30]. Conversely, CSF BAFF levels appeared to be reduced in relapsing MS at clinical onset, particularly in OCGB-positive subjects, suggesting the absorption of this factor by intrathecally recruited B cells from the early phases of the disease [137].

Further research is needed to understand the complex interactions of the BAFF/APRIL system in MS and its potential utility as a prognostic marker.

## Promising biomarkers of B-cell activity under investigation

The search for reliable B-cell activation biomarkers continues, with promising candidates including B-cell-supportive cytokines’ survival receptors shed into the CSF. The actions of cytokines favoring B-cell activity, such as BAFF/APRIL, are controlled by the proteolytic shedding of their receptors (B-cell maturation antigen [BCMA], transmembrane activator and CAML interactor [TACI], and BAFF receptor [BAFFR]) from the surface of B cells, by gamma-secretase [138]. Hoffman and colleagues found that the soluble TACI (sTACI) is elevated in the CSF of pwMS compared to individuals with other neurological diseases without signs of CNS inflammation and strongly correlates with intrathecal IgG synthesis [139]. Additionally, in the field of intrathecal IgG synthesis, IgG effector functions are regulated by the composition of glycans attached to a conserved N-glycosylation site in the crystallizable fragment (Fc). Preliminary results in MS indicated that glycosylation patterns were altered in the CSF, but not in serum [140]. For instance, a lectin-based assay revealed that high CSF IgG galactosylation predicted low disease activity in MS, while CSF IgG galactosylation is reduced in association with disease active progression [141, 142].

Discovering novel MS biomarkers demands extensive methods, such as large-scale proteomics, which has recently emerged as a promising tool for developing disease-specific protein signatures, advancing the understanding of the molecular mechanisms underlying neurological disorders. The proximity extension assay (PEA), combined with next-generation sequencing (PEA-NGS or Olink Explore), merges antibody-based binding with DNA-based signal amplification, allowing highly sensitive and accurate measurement of over 5000 protein levels in small volumes. This technology offers opportunities to identify protein biomarkers with low abundance in body fluids [143, 144]. In a recent study, using Olink Explore to quantify 1463 proteins in CSF and plasma from 143 people with early stage MS and 43 healthy controls, it was found that the top proteins (MZB1, CD79B, CD27, and TNFRSF13B) with the ability to discriminate MS from healthy individuals are all expressed in B cells [145]. In another work on CSF biomarker-based diagnostic tools, other proteins related to expansion and activation of B-cell/plasma cell lineages were shown to effectively distinguish MS from other neurological disorders [146]. Finally, in a comprehensive profiling study of immune activation in the CSF of 118 individuals (58 OCGB + pwMS, 24 OCGB-pwMS, and 36 subjects with other neurological diseases), using PEA for quantifying 92 immune-related proteins, the panel of six CSF immunological proteins with the highest discriminatory power in the comparison between MS and controls comprises Tumor Necrosis Factor Beta (TNFB), a marker of chronic meningeal inflammation which is gaining significant attention in the context of MS. Indeed, TNFB is found to be elevated in the CSF of individuals with relapsing MS exhibiting a high number of cortical lesions and in people with progressive MS with high burden of gray matter demyelination in post-mortem brains, also suggesting the prognostic significance of this protein [58].

## Discussion and future directions

Neurological diseases are characterized by intricate and coexisting molecular pathways, which can be assessed via CSF and blood biomarkers. This molecular dissection of neurological diseases is prompting for etiological and personalized therapy selection [11]. In MS, due to its heterogeneity, the need for objective markers for early diagnosis, subtyping, and prognosis becomes more pressing. Moreover, with the expanding range of DMTs, there is a heightened demand for biomarkers to monitor treatment response and predict adverse effects [147].

The role of B cells as an important driver in MS pathogenesis is nowadays well recognized. It was precisely the discovery of a B-cell activation biomarker, namely CSF-specific OCGBs, several decades ago, as a sensitive diagnostic indicator, and its subsequent clinical implementation, that has stimulated a search for additional fluid biomarkers. Among CSF B-cell activation biomarkers, in the diagnostic setting, markers of intrathecal IgG synthesis are the only ones used in clinical practice so far, playing a crucial role. While the IgG index has limited diagnostic value, OCGBs remain central for supporting and enabling early diagnosis. The κ-index, which is easier to measure and performs comparably to OCGBs, could in the future integrate and/or replace OCGBs determination. Indeed, using both the κ-index and OCGBs can enhance diagnostic specificity, which is particularly useful in atypical cases. From a prognostic perspective, OCGBs are a valid predictor of future disease activity and progression, as is the κ-index, at least in the short to medium term. Moreover, both markers correlate with cognitive impairment, with the κ-index offering the added advantage of being a quantitative marker. Markers of intrathecal IgM synthesis and CSF CXCL13 also hold strong prognostic value, often indicating a more aggressive disease course, potentially surpassing the predictive capabilities of intrathecal IgG synthesis markers. However, their routine use is not yet established in clinical practice due to the limited availability of standardized assays and the need for further validation in large-scale clinical studies (Table 1).

**Table 1:** Diagnostic and/or prognostic value of CSF markers of intrathecal B-cell activation

	Intrathecal IgG synthesis		Intrathecal IgM synthesis		Intrathecal Ig synthesis	B-cell cytokines/chemokines	
Potential application	OCGBs	IgG index	OCMBs	IgM index	κ-index	CXCL13	BAFF/APRIL
DIAGNOSIS	Present in up to 95% of MS cases [53] Included in the 2017 McDonald diagnostic criteria for MS, facilitate an earlier diagnosis [9, 55] with limited specificity (not exclusive to MS) [57] Provide a qualitative, time-consuming, and operator-dependent output [46] **Currently, the most established and widely used fluid marker in MS diagnosis**	High CSF IgG index reported in 70%–80% of MS cases [67] Less sensitive than OCGBs [67]	Intrathecal IgM synthesis reported in 20–55% of MS cases [42, 70]		As opposed to OCGBs, it provides a quantitative, automated, non-operator-dependent output [94–107] Diagnostic accuracy similar to OCGBs [99, 100] Alternative or complementary to OCGBs in the appropriate clinical context [101–107] **Its inclusion proposed in the next revision of MS diagnostic criteria** [101–103]	Elevated in MS CSF [116, 117] Limited specificity (not exclusive to MS) [119]	Elevated in MS CSF [133] Limited specificity (not exclusive to MS) [134–136]
PROGNOSIS	Predict conversion from RIS [61] and CIS [54, 56] to MS Predict important disability milestones and MS progression [56, 60, 62] Correlate with cognitive impairment [63] and with higher cortical lesion load on MRI [64]	Predicts the risk of higher disability and MS progression [68] Associates with more severe gray matter atrophy [69]	Associates with heightened susceptibility to relapses (surpassing the risk associated with OCGBs) [42, 81, 82] Associates with higher disability [83] and shorter time to progression onset [84] Correlates with a more inflammatory phenotype in pwMS presenting a primary progressive course [85]		Predicts the risk of subsequent relapses [108, 110] and of new MRI lesions in early MS [111] Predicts the risk of PIRA [109] Correlates with worse cognitive performance [112, 113], independently of MRI measures [113]	Predicts the risk of subsequent relapses or new/enhancing MRI lesions (better than OCGBs) [121–123] Correlates with higher EDSS and MS progression [121] Increased CSF levels in naïve pwMS with high cortical lesion load on MRI [30] Increased expression in the meninges and CSF of post-mortem progressive MS [30]	Increased CSF levels in naïve pwMS with high cortical lesion load on MRI [30]

*Abbreviations.* OCGBs: IgG oligoclonal bands; OCMBs: IgM oligoclonal bands; CXCL13: C–X–C motif chemokine ligand 13; BAFF: B-cell activating factor; APRIL: A proliferation-inducing ligand; MS: multiple sclerosis; CSF: cerebrospinal fluid; RIS: radiologically isolated syndrome; CIS: clinical isolated syndrome; pwMS: people with multiple sclerosis; MRI: magnetic resonance imaging; EDSS: Expanded Disability Status Scale; PIRA: progression independent of relapse activity

The search for robust fluid B-cell activation biomarkers continues; long-term studies involving large cohorts are essential to validate biomarker candidates and translate them into clinical practice. While most of the studies are performed on CSF, since its proximity to the CNS makes it an ideal matrix for biomarker investigation, there is an urgent need, especially for disease monitoring purposes, to develop serum biomarkers that are more accessible and non-invasive. This review specifically addresses CSF B-cell activation biomarkers, but dedicated investigations on blood-based biomarkers are mandatory in the near future [148]. Finally, in the exploration of B-cell activation biomarkers for optimizing clinical decision-making and treatment strategies, a forward-looking perspective consists in elucidating the specific B-cell repertoire that contributes to disease processes and developing reliable tools for monitoring these populations and their impact on immune responses. The diverse outcomes observed with anti-CD20 and BAFF/APRIL system-targeting therapies underscore the necessity of better understanding B cells' dual role in concurring to immunologically mediated diseases and regulating autoimmunity. Further studies to examine how the clinical course and response to treatment relate to the levels and activity of key B-cell subpopulations and molecules involved in B-cell activation and regulation could definitively establish and characterize the role of B cells in MS pathogenesis, providing opportunities for more targeted MS treatments, eventually addressing pathogenic mechanisms while preserving protective B-cell functions.
